# Polycarbonate Plastics and Human BPA Exposure: Urinary Levels Rise with Use of Drinking Bottles

**DOI:** 10.1289/ehp.117-a406b

**Published:** 2009-09

**Authors:** Victoria McGovern

**Affiliations:** **Victoria McGovern**, based in Durham, North Carolina, has written for *EHP* since 2000. She is a member of the National Association of Science Writers

Public and scientific concerns about exposure to bisphenol A (BPA) have risen in the last few years, with Canada and some U.S. states and cities banning BPA from polycarbonate baby bottles and other products sold for use by infants and children. Despite these concerns, little is known about whether the use of polycarbonate food or beverage containers actually contributes to BPA body burden in people. A new study of human exposure to BPA from drinking containers now shows that study participants’ urinary concentrations of the molecule increased by two-thirds after they used polycarbonate drinking bottles for 1 week **[*EHP*** **117:1368–1372; Carwile et al.]**.

Rodent studies have associated prenatal and neonatal exposure to BPA with early onset of sexual maturation, reproductive tract lesions, and altered development of the mammary gland, among other reproductive abnormalities. However, limited information is available on human health effects. Nevertheless, human exposure to BPA is widespread: the chemical was detected in the urine of more than 92% of the participants aged 6 years and older in the 2003–2004 National Health and Nutrition Examination Study (NHANES).

Not all polycarbonate plastics contain BPA, but nearly three-quarters of the BPA used in the United States in 2003 went into the manufacture of this one material. The hard, nearly shatterproof plastic is widely used in drinking bottles, baby bottles, and non-food uses ranging from eyeglasses to labware. Earlier studies of polycarbonate drinking containers containing BPA have shown that under normal use—washing, rinsing, and exposure to high temperatures or to alkali or acid solutions—the plastic can degrade and release small amounts of the constituent chemical.

BPA is believed to be rapidly metabolized and eliminated. Therefore, in the current study, 77 college students aged 18–22 underwent a weeklong “washout” to minimize any preexisting BPA load that could have arisen from the use of polycarbonate drinking bottles. During the washout, participants were instructed to drink any cold beverages only from stainless steel bottles and to avoid drinking water from the polycarbonate dispensers in the college dining halls. After the washout, the group switched to drinking cold drinks only from 2 new researcher-provided polycarbonate bottles for 1 week. Exposure to other BPA sources was not controlled; thus, the study yielded a conservative estimate of the potential for BPA exposure via polycarbonate drinking bottles.

Comparison of urine samples collected throughout the study showed that after using polycarbonate bottles for 1 week, participant’s mean urinary BPA concentrations increased by more than two-thirds to 2.1 μg/L, compared with the mean of 2.6 μg/L observed in the NHANES 2003–2004 study. The authors anticipate higher urinary BPA concentrations would result from drinking hot beverages stored in the same bottles.

